# Infectious Diseases Institute at Makerere University College of Health Sciences: a case study of a sustainable capacity building model for health care, research and training

**DOI:** 10.4314/ahs.v22i2.3S

**Published:** 2022-08

**Authors:** Damalie Nakanjako, Barbara Castelnuovo, Nelson Sewankambo, Tom Kakaire, Richard L Brough, Elly T Katabira, David L Thomas, Thomas C Quinn, Robert Colebunders, Warner C Greene, Allan R Ronald, Alex Coutinho, Keith McAdam, David Serwadda, Fred Wabwire-Mangen, Edward Katongole-Mbidde, Philippa Musoke, Moses Joloba, Henry McKinnell, Moses Kamya, Harriet Mayanja-Kizza, Yukari C Manabe, Andrew Kambugu

**Affiliations:** 1 School of Medicine, Makerere University College of Heath Sciences, Kampala, Uganda; 2 Infectious Diseases Institute, Makerere University College of Heath Sciences, Kampala, Uganda; 3 Former Executive Director, Infectious Diseases Institute, Kampala, Uganda; 4 Division of Infectious Diseases, Department of Medicine, Johns Hopkins University School of Medicine; 5 Division of Intramural Research, National Institute of Allergy and Infectious Diseases, National Institutes of Health, Bethesda, MD, USA; 6 Global Health Institute, University of Antwerp, Antwerp, Belgium; 7 Gladstone Institute of Virolology and Immunology, University of California, San Francisco, USA; 8 University of Manitoba, 99 Wellington Crescent, Winnipeg Manitoba, R3M0A2, Canada; 9 School of Public Health, Makerere University College of Heath Sciences, Kampala, Uganda; 10 Former Director, Uganda Virus Research Institute, Kampala, Uganda; 11 School of Biomedical Sciences, Makerere University College of Heath Sciences, Kampala, Uganda; 12 Former Chairman, Moody's corporation, Former Chairman and CEO Optimer Pharmaceuticals and Former Chairman and CEO Pfizer Inc

**Keywords:** Capacity building, public-private partnership, global epidemic response

## Abstract

The Infectious Diseases Institute (IDI), established in 2001, was the first autonomous institution of Makerere University set up as an example of what self-governing institutes can do in transforming the academic environment to become a rapidly progressive University addressing the needs of society This paper describes the success factors and lessons learned in development of sustainable centers of excellence to prepare academic institutions to respond appropriately to current and future challenges to global health.

Key success factors included a) strong collaboration by local and international experts to combat the HIV pandemic, along with b) seed funding from Pfizer Inc., c) longstanding collaboration with Accordia Global Health Foundation to create and sustain institutional strengthening programs, d) development of a critical mass of multi-disciplinary research leaders and managers of the center, and e) a series of strong directors who built strong governance structures to execute the vision of the institute, with subsequent transition to local leadership.

**Conclusion:**

Twenty years of sustained investment in infrastructure, human capital, leadership, and collaborations present Makerere University and the sub-Saharan Africa region with an agile center of excellence with preparedness to meet the current and future challenges to global health.

## Introduction

The Infectious Diseases Institute (IDI) at Makerere University was established in 2001 through a public-private partnership at Makerere University Faculty of Medicine in Uganda, involving a group of fourteen like-minded and foresighted Ugandan and North American clinician scientists named the Academic Alliance for AIDS Care and Prevention in Africa (AA), Pfizer Pharmaceuticals, and the Government of Uganda 1, 2. Key leaders of this Alliance included Dr. Merle Sande*, President of the AA Foundation and Chair of Medicine, University of Utah, Nelson Sewankambo, Dean of the Faculty of Medicine at Makerere University, and Henry “Hank” McKinnell, Chief Executive Officer (CEO) of Pfizer. These leaders and the Academic Alliance realized that as anti-HIV drugs became more widely available in sub-Saharan Africa, it was essential to create a strong cadre of well-trained doctors, nurses, and laboratory workers who could not only administer these drugs and care for HIV-infected people but also could train others to deliver an equally high level of care. They further realized that building a center of excellence in East Africa at Makerere had the potential to produce a multiplier effect not only regionally but perhaps across the entire continent of Africa. A vision of the IDI crystallized; a center of excellence squarely focused on training, clinical care and research.

The IDI was envisioned to train tens of thousands of medical professionals who in turn would each train hundreds who would diagnose, treat, and care for the tens of millions who needed help. A bold ambition indeed. Part of that vision was to reject the idea of “good enough for Africa.” Everything the IDI did from building construction to clinical research, to the laboratory, to patient care would be “world class.” Moreover, the goal was African-built and African-led, an even bolder ambition. While many from other continents contributed to the IDI's success, that bold ambition has been realized. The key success factors and lessons learned with the IDI, as described in this paper by several IDI founders, provide an informative roadmap to create strategic partnerships with academic institutions in low- and middle-income countries (LMIC) to respond to current and future challenges in global health.

Key success factors for development of a self-sustaining and productive center of excellence, such as IDI, at an academic institution in LMIC are described below

**a) Strong collaboration by local and international experts to combat the HIV pandemic:** The AIDS crisis hit San Francisco hard in the early 1980s. As Chief of Medicine at the San Francisco General Hospital Dr. Sande, often described as an “impatient visionary”, together with his colleagues created the San Francisco model of AIDS care involving specialized inpatient and outpatient facilities aimed at compassionate and comprehensive care. Through a unique public-private partnership, Dr. Sande played a key role in creating the Gladstone Institute of Virology and Immunology, a research center dedicated to fundamental studies of the virus. With San Francisco in mind, Merle had a vision for what could be accomplished at Makerere University. He forged a strong partnership with Dean Sewankambo and Pfizer's Hank McKnnell that ultimately led to the creation of the IDI. He was aided in this quest by his creation of the Academic Alliance, comprised of a strong and committed collection of physicians and scientists from Africa, North America and Europe. Among his many honors, Merle treasured the Presidential Distinguished Service Award for Contribution to Health Care in Uganda. IDI will always be indebted to Dr.Sande's vision, tenacity, and leadership that were key to its creation.

The partnership of IDI with the Accordia Global Health Foundation (the successor to the Academic Alliance Foundation) grew exponentially stronger as IDI launched operations in its new building. Accordia's mission was to overcome the burden of infectious diseases in Africa by building centers of excellence and strengthening medical institutions. Accordia was committed to the creation of African owned and African led permanent centers that develop health leadership, skills, and knowledge to create generations of healthy children and adults. Key personnel at Accordia included Warner C. Greene MD, PhD, Accordia's President and member of the Academic Alliance, Carol Spahn, Executive Director, and Kelly Willis, Vice President and Director of Programs. Accordia worked closely with IDI to build vibrant programs. A few examples of Accordia's programs at IDI include a Visiting Professor-In-Residence series, the Sewankambo Scholars Program, an annual Scientific Advisory Board review involving international leaders in infectious disease, Gates funding to better understand how to train mid-level health workers to care for HIV infected people, and new funding for recruitment of IDI leadership and construction of IDI's second building. Accordia also held annual conferences bringing together 13 leading centers in Africa to share lessons learned and the best approaches to accomplish their mission of building stronger organizations more effectively for the delivery of healthcare. IDI was a center point of these discussions and steadily grew as a sustainable and effective model for all of Africa. Accordia merged with Africare in 2017.

**b) Purposeful public-private partnerships and cooperate responsibility to combat global health challenges:** By mid-2000, Hank McKinnell, Pfizer's then 30-year veteran and CEO was selected by Pfizer's Board of Directors to become Chairman/CEO early in 2001. Hank's belief was that companies' success needed to be measured by the trust and respect of all stakeholders not just shareholders. While Pfizer had served its shareholders extremely well up until that time and was one of the most valuable companies in the world, Pfizer and the pharmaceutical industry generally were far from the most respected. In 2000, Pfizer's chairman/CEO was burned in effigy at the World's AIDS Conference in Durban, South Africa for “putting profits before patients.” Hank knew that criticism was unfair and set as one of his goals earning back public trust. Hank met with Pfizer's infectious disease advisory board of 20 of the world's most respected academic researchers (the IDAB). Their advice was “*Pfizer didn't needto tell their story better, they needed to have a better story to tell”.*

Out of that meeting grew the Diflucan (Fluconazole) program at the IDI. Hank realized health care in Africa was delivered in several different ways. The private market was separate from government-run healthcare in many countries and Pfizer could provide Diflucan, life-saving medicine for patients with HIV and cryptococcal meningitis at no charge to the government while maintaining private sector sales. The Diflucan program was hugely successful and saved many lives.

**c) Bold ambitions and visionary leadership:** Hank also served on President George W. Bush's Presidential Advisory Council on HIV/AIDS (PACHA) and knew President Bush was committed to providing help to Africa in dealing with the triple threats of HIV/AIDS, Malaria, and Tuberculosis. Hank further realized that medical aid was not like food aid. Medicines could not just be handed out from the back of a truck. Supported by the advice of Pfizer's IDAB, the NIH, the Center for Disease Control and prevention (CDC), and in particular Dr. Merle Sande and Hank met with the Presidents of South Africa, Kenya, and Uganda, seeking a public sector partner for what Pfizer was about to do. “The clear winner was President Museveni and the government of Uganda”, said Hank. That decision was strongly supported by Dr. Merle Sande who as a young clinical research doctor had done pioneering work in Uganda and had made many friends there. Dr. Sande brought on board Dr. Nelson Sewankambo.

**d) Autonomy within a reputable University:** Governance of an autonomous institute (wwwidi-makerere. com) through an independent IDI Board allowed IDI to benefit from some University resources including but not limited to land, technical expertise, established academic programs and the University brand. IDI is headed by an Executive Director (ED) with the support of a 14 -member senior management team (a head and deputy head for 6 programmes and 2 sub-programmes plus a head and deputy head for 3 support functions). The ED reports to a Board with 12 members who are selected on their own merit for specific competencies that they bring to the Board. The Board provides strategy and policy oversight and meets a minimum of 3 times a year to review progress and to provide recommendations for progressing IDFs mission. The Board has a standing Audit & Finance Committee, with other ad hoc committees such as the nominations committee formed as and when needed. Board members serve for a four-year renewable term and are rotated away at the end of the second term to create room for new board members. The Board nominations committee scouts globally for potential candidates for nomination to the IDI board and to the ED position. The Board reports annually to the Annual General Meeting (AGM) of members. The members, who are the apex governance body of IDI are the University Vice Chancellor and University Secretary; and through the AGM they provide an annual high-level review of IDFs financial position and its progress in achieving its mission. The members also approve and make the final appointment of new nominees to the Board and ED positions.

By having a full-time executive director and independent financial management and procurement systems, the IDI has attained a reputable efficiency to implement multiple projects, with presence in different parts of the country. Through its independent recruitment processes, IDI is able to hire technical and support staff as core staff and research staff, as and when required. IDI staff have increased from 28 core staff in 2004 to 2058 in 2022 (Table 2). Results from multiple studies are published in reputable peer-reviewed journals 4–8, with over 940 peer-reviewed publications over the past two decades. These results have led to programmatic improvements and improved outcomes of HIV treatment and management of co-infections country-wide; thereby amplifying the college of health sciences' mandate to respond to societal needs.

**Table 2:** Cumulative increase of core and project staff at the Infectious Diseases Institute Makerere University between September 2004 and April 2022

Year	Core staff	Projects staff	Grand total
**Sep 2004**	28	0	28
**Aug 2005**	210	54	264
**Dec 2006**	230	38	268
**Jun 2007**	205	107	312
**Dec 2008**	141	111	252
**Dec 2009**	156	219	375
**Jun 2010**	165	294	459
**Jun 2011**	176	501	677
**Jun 2014**	178	532	710
**Jun 2015**	152	665	817
**Jun 2016**	154	748	902
**Sept 2016**	154	731	885
**Dec 2016**	157	762	919
**Mar 2017**	163	746	909
**Jun 2017**	188	856	1,044
**Sept 2017**	189	817	1,006
**Dec 2017**	200	1,035	1,235
**Jan 2018**	209	1,135	1,344
**Dec 2019**	217	1144	1361
**Dec 2020**	227	1389	1616
**Dec 2021**	238	1646	1884
**Apr 2022**	261	1797	2058

**e) Seed funding:** The Pfizer initial investment in the IDI greatly facilitated the establishment of infrastructure, governance and management systems. Apart from direct in-kind expenditure, cumulative annual Pfizer unrestricted grants totaled about $25,000,000 from FY2005/2006 (when IDI was transferred to Makerere University and started maintaining its own accounts to FY 2011/2012). This generous support laid a firm foundation that has enabled the institute to grow its programmatic areas: Research, Training, and Prevention, Care & Treatment, and subsequently the outreach program (later renamed Health Systems Strengthening), Laboratory Services, and the more recently added Global Health Security (GHS), Academy for Health Innovation, and HIV prevention research site (IDI Kasangati).

**f) Capacity building and mentorship for science leadership:** The IDI attracted several capacity building programs to educate and train scientists who are able to innovate local solutions to local problems of global importance including HIV/AIDS, tuberculosis, malaria, hepatitis B, Ebola, and more recently COVID-19. Overall, IDI has contributed to training of 31 PhD and 63 Masters' degree graduates, 12 post-doctoral scientists and over 563 trainees (different cadre) in short courses including scientific writing and research project management (Table 1). Through capacity building programs including the initial two-year fellowship in Infectious Diseases and the five-year Sewankambo clinical scholarship programs, the institute developed its leaders including the current executive director, Andrew Kambugu, the current Principal of MakCHS, Damalie Nakanjako, and others who are leading several departments and research groups at the college of health sciences 3. Through co-mentorship with scientists in collaborating academic institutions in the USA and Europe several doctoral trainees, post-docs and fellows have been supported at the IDI which is now an international training site for Fogarty training programs (D43 and U54 funded by the National Institute of Health, USA) and European and Developing Countries Clinical Trials Partnership (EDCTP), among others.

**Table 1:** Training of various health cadre in HIV, malaria and TB prevention treatment and diagnosis as per December 2021

Cadre	Trainee	Percentage
Clinical Officers	5,291	10.5%
Medical Officer/Doctor	3,603	7.1%
Nurses/ Midwives	11,184	22%
Pharmacist / Dispensers	548	1.5%
Nursing Assistants	4,569	9%
Laboratory staff	8,437	17%
Records staff	2,165	4%
Counsellors	1,978	4%
M&E Officers	733	1.1%
Stores Assistant/Store keeper	186	0.4%
Medical Students	367	1.4%
Other	11,257	22%

**Total**	**50,318**	**100%**

**g) Strengthening laboratory capacity for clinical care and translational research:** Providing quality health care was challenged by limited capacity for laboratory testing 9. Through partnership with John Hopkins University (JHU), a core lab facility was established and certified by the American College of Pathologist (CAP), to provide high quality tests to support patient care and research 10–13. A translational research lab was established, separate from the Core lab, in order to allow trainees to pursue lab-based science and to develop new tests to support higher impact research. Through the College of Health Sciences collaborations with the Gladstone Institute at University of California San Francisco (UCSF), a translational research laboratory was expanded by equipping a larger laboratory space that was available under the Department of Obstetrics and Gynecology. The translational laboratory hosts several masters', doctoral and post-doctoral trainees who conduct translational research that was previously only conducted abroad. The translational research laboratory provides opportunities for innovation of new assays by the Microbiology, Mycology, Pharmacokinetics, Immunology and Molecular biology teams, with the aim of optimizing the innovations for routine patient care in the CAP-certified laboratory. There is still a need to build capacity for research with animal models to promote further understanding of genetics and pathogenesis of human diseases and related innovative therapies in sub-Saharan Africa.

**Figure 2:**
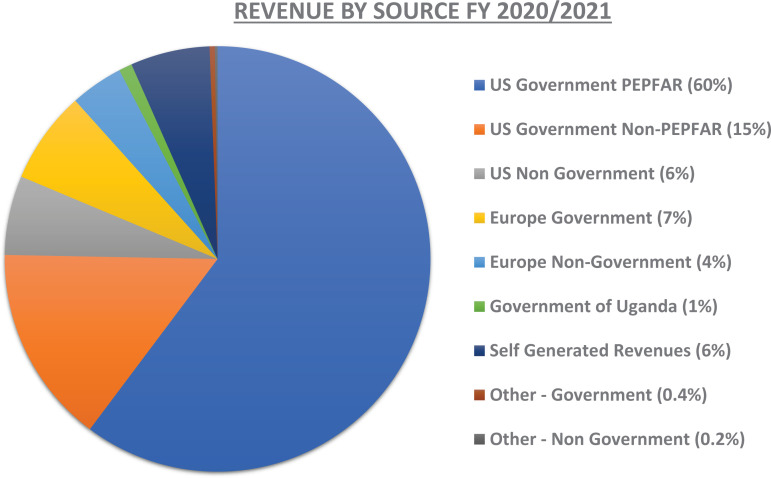
Revenue by source of funding for the Infectious Diseases Institute at Makerere University in fnancial year 2020/2021

**h) Grants management systems and diversification of funding:** A critical element in facilitating sustainability of the Institute was the establishment of a grants management unit during the initial period of Pfizer's financial investment. This unit was skilled with pre-and post-award grants management capacity and by the time the funding commitment from Pfizer concluded in 2012, IDI was raising an annual income of about $20,000,000 through multi-year grants. Currently, the IDI operates an annual budget of approximately $60,000,000 from various funders as shown in Figure 2. The funding portfolio has been significantly diversified and includes United States government agencies United States Agency for International Development (USAID), Centers for Disease Control and Prevention (CDC), United States Department of Defense (DoD), and the National Institute of Health (NIH), the European and Developing Countries Clinical Trials Partnership (EDCTP), the Flemish Interuniversity Council for University Development Cooperation (VLIR-UOS), the United Kingdom Fleming Fund, private foundations (such as the Elma Foundation and the Johnson and Johnson Corporate Citizenship Trust), Gates Foundation, Wellcome Trust, the Government of Uganda, and several investigator/program-driven projects. A reputable efficient grants and finance management framework has provided systems that support research by individual scientists, trainees, research groups and multi-disciplinary consortia.

**i) Strategic leadership with transition to local leadership to promote sustainability:** The IDI has been led by a series of internationally reputable directors including Keith McAdam, an Emeritus Professor of Tropical Medicine and founding director (2004–2007), followed by Dr Alex Coutinho who had previously served as director of the AIDS Support Organisation (the largest AIDS support organization in sub-Saharan Africa) and recipient of the 2013 prestigious Hideyo Noguchi Africa prize for outstanding achievement in medical research and services in Africa. Subsequently leadership was transitioned to Richard Brough who had worked as head of strategic planning for nine years and had led IDI's first and second five-year strategic plans including the establishment of a strong grants management systems. The IDI directorship was later taken over by Andrew Kambugu, the institute's first Infectious Disease fellow, who had been mentored at the institute as the head of the prevention, care and treatment program for seven years and subsequently as head of research. The mentorship, capacity building in leadership, and strategic planning have produced admirable institutional continuity and sustainable leaders. The IDI is currently implementing its third five-year strategic plan.

**The impact of academic research and programmes at IDI Research to improve patient care:** The programme is based on priority research questions for Africa, taking advantage of partnerships to build capacity in key focal areas such as: opportunistic infections including TB and Cryptococcus, and clinical pharmacology and pharmacokinetics 5, 14–18. Through partnership, IDI's HIV prevention clinical research has contributed to evidence leading to global drug approval for HIV prevention and revised protocols for use with HIV treatment and management of discordant couples 19, 20.

**Figure 1:**
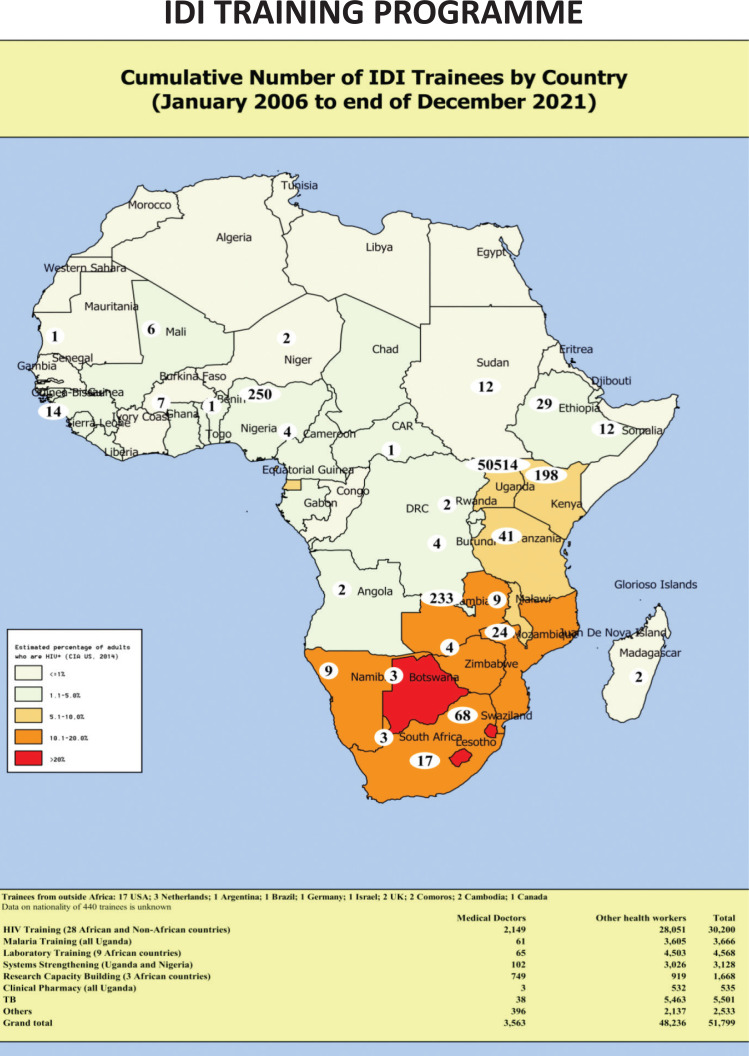
Cumulative number of IDI trainees by country (January 2006 to end of December 2021)

**National and Regional influence:** IDI has trained over 51,799 health workers from Uganda and other African countries in infectious disease prevention and treatment, laboratory skills and various aspects of systems strengthening (Figure 1). The training department provides systematic ongoing distance support, eHealth approaches and a free call-in service for both patients and health care providers, and has informed government policies and guidelines, including introduction of ‘Test & Treat’ for HIV-infected patients. In addition, IDI supports nation-wide health systems strengthening (HSS) with programmes that address key functions across the World Health Organization (WHO) health system pillars (such as HR management) using an HIV platform. IDI is currently one of the lead implementing partners for the US President's Emergency Plan for AIDS Relief (PEPFAR)/US Centers for Disease Control and Prevention (CDC)/Ministry of Health. The institute currently supports care and treatment in 14 districts in the Kampala and West Nile Regions, covering over 270,000 people living with HIV (approximately 20% of all PLHIV in Uganda). Notably, IDI was selected by the US National Institutes of Health (NIH) to host the African Centre of Excellence in Bioinformatics & Data Sciences (ACE) which positions IDI to take part in the global data science revolution to advance Computational Biology and big data analysis concepts to manage current and future epidemics.

**Table:** 

Funding Agency	Amount in USD (Percentage)
US Govt PEPFAR	35,910,591 (60%)
US Govt Non PEPFAR	8,892,993 (15%)
US Non Govt	3,836,368 (6%)
Europe Govt	3,989,939 (7%)
Europe Non Govt	2,629,323 (4%)
Govt of Uganda	445,442 (1%)
Self-Generated Revenues	3,535,637 (6%)
Other - Government	248,915 (0.4%)
Other - Non Government	114,828 (0.2%)
**Total**	**59,604,037 (100%)**

**South-South collaborations:** IDI has various ongoing relationships with over 10 African institutions. These include University of Zimbabwe, Moi University, Ifakara Health Institute, University of Cape town, University of the Witwatersrand, and Neurologic Association of South Africa, which are either subgrantees or co-implementers of various projects and the East, Central and South African College of Physicians (ECSACOP) for which IDI hosts and provides fiscal management services. Notably, IDI is a fiscal management and Organizational Development founding Partner of Afrehealth, a Pan-African health inter-professional and multi-disciplinary organization established in 2016 with US NIH support in partnership with IDI. The Afrehealth network, whose Secretariat is In Kwame Nkrumah University of Science and Technology (KNUST) in Ghana has over 500 individual members (26 countries) and 46 academic institutional members from 18 countries (across all geographic and linguistic regions of Africa). IDI co-implements a number of projects with Afrehealth. Similarly, IDI was selected by the Africa Union and, Africa CDC with the support of MasterCard Foundation to lead the Implementation Science pillar of the Saving Lives and Livelihoods (SLL) Programme – an unprecedented $1.3 bn commitment for the African continent by Mastercard Foundation. Through its Program for Research on Vaccine Effectiveness (PROVE), IDI will support the Africa CDC to estimate the real-world effectiveness of COVID-19 vaccination on the entire African continent, understand the barriers and enablers of COVID-19 vaccination in Africa and to evaluate the Impact of COVID-19 vaccination on national health systems. Implementation of country PROVE grants shall inform COVID-19 vaccination rollout programmes across the African continent.

## Lessons learned

- Strategic partnership of leading scientists in Infectious diseases supported the national and regional response to , 375 Greenbrier Drive, Charlottesville, VA, 22901 the HIV pandemic
- Public-private partnerships with academic institutions together with significant seed funding and political will provide opportunities for capacity building for health care and research in resource-limited settings
- Training and mentorship of future leaders is relevant for growth and sustainability of productive academic centers of excellence
- Rapid growth of human resource to meet demands of the expanding core and research functions of the institute requires robust and dynamic human resource systems.
- Wholistic development of physical, human, and systems infrastructure, within a stable training environment prepares academic institutions to lead innovative approaches to emerging challenges. For example, through the IDI and other clinical, laboratory and public health units at MakCHS, Makerere University was able to provide a formidable support to the national response to the COVID-19 pandemic.
- Investments in strong research infrastructure (grants management systems, regulatory compliance, biostatistics and data management systems, longitudinal cohorts, research laboratories, and Information Communication Technology) to build translational research are all critical for productive academic research to impact community for better health.
- Robust governance structures are critical to the efficiency, success and impact autonomous centers of excellence within academic institutions.
- At the time, 2001, the HIV disease burden was overwhelming therefore illness/disease care became the primary goal. Although Public Health and preventive care is the most effective, strategic, and least costly way to promote better health and save lives, many people tend to wait until they are disabled, ill, or near the end of life before realizing that the illness/disease could have been prevented/avoided.
- Within the IDI, Makerere University hosts: a) Clinical and basic scientists at all levels to attract funding for research and training programs, b) strong local and international collaborations and networks, c) strong and reputable research support infrastructure, d) state-of-the art physical infrastructure with research laboratories and data systems to understand disease epidemiology, e) ability to conduct translational science locally, and f) a global health security program.
- Through collaborative efforts in an environment that promotes academic freedom, many people contributed to development of the IDI at Makerere University and many were able to walk away and not ‘take credit’ yet all remain so proud of what has been accomplished.
- Makerere University has learned great lessons from IDI and its development, and has gone ahead to approve the creation of over ten other progressive self-governing institutes of the University to provide similar supportive environments that motivate staff to be very productive. In this regard Makerere is providing leadership and direction to other universities on the continent on how to be innovative and do business differently.

*“The creation of IDI opened a new chapter to the University regarding how it should do business differently. If the University takes the lessons from IDI at heart, Makerere would be a vey different place in the next 10 years and I hope I can live to see that transformation”* said Nelson Sewankambo, one of the fourteen founder members of the Academic Alliance

*“IDI is continuing to transform Makerere, the health system and policy landscape, and impacting peoples' lives, the friends of IDI* ”, he added.

## Conclusion

Twenty years of sustained investment in infrastructure, human capital, leadership, and collaborations presents Makerere University and the sub-Saharan Africa region with an agile centre of excellence with preparedness to meet the current and future challenges to global health. The commitment of large startup funds from Pfizer, plus ongoing annual support by the Government of Uganda, Makerere University institutional support, as well as numerous local and international collaborations have collectively led to the sustained productivity of the IDI in national and regional response to emerging infectious disease challenges.
